# Nanoroughened adhesion-based capture of circulating tumor cells with heterogeneous expression and metastatic characteristics

**DOI:** 10.1186/s12885-016-2638-x

**Published:** 2016-08-08

**Authors:** Weiqiang Chen, Steven G. Allen, Ajaya Kumar Reka, Weiyi Qian, Shuo Han, Jianing Zhao, Liwei Bao, Venkateshwar G. Keshamouni, Sofia D. Merajver, Jianping Fu

**Affiliations:** 1 Department of Mechanical Engineering, University of Michigan, Ann Arbor, MI 48109 USA; 2 Department of Mechanical and Aerospace Engineering, New York University, New York, NY 10012 USA; 3 Program in Cellular and Molecular Biology, University of Michigan, Ann Arbor, MI 48109 USA; 4 Medical Scientist Training Program, University of Michigan, Ann Arbor, MI 48109 USA; 5 Department of Internal Medicine, University of Michigan, Ann Arbor, MI 48109 USA; 6 School of Advanced Engineering, Beihang University, Beijing, 100191 China; 7 University of Michigan Comprehensive Cancer Center, University of Michigan, Ann Arbor, MI 48109 USA; 8 Department of Biomedical Engineering, University of Michigan, Ann Arbor, MI 48109 USA; 9 Department of Cell and Developmental Biology, University of Michigan, Ann Arbor, MI 48109 USA; 10 Michigan Center for Integrative Research in Critical Care, University of Michigan, Ann Arbor, MI 48109 USA

**Keywords:** Circulating tumor cells, Adhesion, Microfluidics, Metastasis, Breast cancer, Lung cancer

## Abstract

**Background:**

Circulating tumor cells (CTCs) have shown prognostic relevance in many cancer types. However, the majority of current CTC capture methods rely on positive selection techniques that require a priori knowledge about the surface protein expression of disseminated CTCs, which are known to be a dynamic population.

**Methods:**

We developed a microfluidic CTC capture chip that incorporated a nanoroughened glass substrate for capturing CTCs from blood samples. Our CTC capture chip utilized the differential adhesion preference of cancer cells to nanoroughened etched glass surfaces as compared to normal blood cells and thus did not depend on the physical size or surface protein expression of CTCs.

**Results:**

The microfluidic CTC capture chip was able to achieve a superior capture yield for both epithelial cell adhesion molecule positive (EpCAM+) and EpCAM- cancer cells in blood samples. Additionally, the microfluidic CTC chip captured CTCs undergoing transforming growth factor beta-induced epithelial-to-mesenchymal transition (TGF-β-induced EMT) with dynamically down-regulated EpCAM expression. In a mouse model of human breast cancer using EpCAM positive and negative cell lines, the number of CTCs captured correlated positively with the size of the primary tumor and was independent of their EpCAM expression. Furthermore, in a syngeneic mouse model of lung cancer using cell lines with differential metastasis capability, CTCs were captured from all mice with detectable primary tumors independent of the cell lines’ metastatic ability.

**Conclusions:**

The microfluidic CTC capture chip using a novel nanoroughened glass substrate is broadly applicable to capturing heterogeneous CTC populations of clinical interest independent of their surface marker expression and metastatic propensity. We were able to capture CTCs from a non-metastatic lung cancer model, demonstrating the potential of the chip to collect the entirety of CTC populations including subgroups of distinct biological and phenotypical properties. Further exploration of the biological potential of metastatic and presumably non-metastatic CTCs captured using the microfluidic chip will yield insights into their relevant differences and their effects on tumor progression and cancer outcomes.

**Electronic supplementary material:**

The online version of this article (doi:10.1186/s12885-016-2638-x) contains supplementary material, which is available to authorized users.

## Background

While progress has been made on the prevention and treatment of primary cancers, metastases to distant sites remain a major clinical challenge and the main cause of death for the majority of cancer patients [1]. Thus attention has shifted toward a better understanding of the metastatic process in order to address the mortality of patients with metastatic lesions. The spread of cancer systemically relies upon the critical step of the hematogenous spread of cancer cells [2]. These circulating tumor cells (CTCs) in the bloodstream are shed from primary and metastatic lesions and are believed to be key agents in the metastatic process [2–4]. Therefore, capturing CTCs is not only important to understand the determinants of the metastatic fate of cancer cells, but also directly yields clinically relevant information as studies on CTCs have shown a general, but not complete, negative association between CTC counts and clinic outcomes [5–7]. The challenge being as a tumor progresses down the metastatic cascade, cancer cells are known to express diverse molecular phenotypes in a dynamic fashion, which complicates the isolation of CTCs for further study [6, 8–13]. Moreover, other cells such as fibroblasts and non-cancerous epithelial cells are also shed into the circulation further complicating the identification of the true potentially metastatic cells.

The most widely used methods for CTC capture have relied upon tumors’ cell of origin and utilized antibodies against tissue specific surface markers, notably epithelial cell adhesion molecule (EpCAM), which is expressed by epithelial cells [14–21]. However, numerous studies have demonstrated that the EpCAM antibody-based positive selection method is imperfect, as EpCAM expression on cancer cells varies not only from patient to patient but also within the same patient over time [6, 8, 9, 11, 12]. Furthermore, studies have demonstrated that epithelial-specific markers are selectively partially or completely down-regulated over the course of tumor dissemination through the epithelial-to-mesenchymal transition (EMT) [10, 13]. Other CTC capture methods utilize size-based selection, as cancer cells are believed to be generally larger than hematopoietic and other shed cells and thus amenable to filtration or centrifugation. However, CTCs of various sizes, including some smaller than leukocytes, have been reported recently [22–24]. The major challenge of CTC isolation is the extreme rarity of CTCs, even in patients with advanced cancer. This is especially evident when using negative selection techniques which deplete the undesired leukocyte population using antibodies against CD45, a leukocyte cell surface marker. Thus, because of the rarity of CTCs, it is difficult for negative selection techniques alone to achieve satisfactory yields for CTC capture [25, 26].

Along the complex and dynamic progression through the metastatic cascade there is however an important point of convergence. The intravasation step into blood vessels by certain cancer cells within a tumor is a mechanically focused process by its very nature, and only those cells capable of behaving in a precise biomechanical way will successfully enter the bloodstream as live cells [27–29]. The mechanical phenotype of a cancer cell results from the integration of multidimensional and heterogeneous factors such as cell intrinsic genetic expression and epigenetic regulation and cancer cell extrinsic signals from cytokines, growth factors, and extracellular matrix proteins as well as interactions involving non-cancerous immune and stromal cells [27, 30]. Given these complex inputs into the cancer cell phenotype, we set out to develop a method for CTC capture that does not rely upon any one single facet of this complex set of determinants, such as surface marker expression, but instead relies upon an output that reflects the integration of the multitude of signaling pathways experienced by a spreading cancer cell.

To this end, we developed a method that captures CTCs based on their differential capability to selectively adhere to a nanoroughened glass surface as compared to normal blood cells. In our prior work [31], we described that a nanorough glass substrate generated by reactive-ion etching (RIE) without any positive-selection antibodies exhibits significantly improved cancer cell capture efficiency owing to enhanced adherent interactions between the nanoscale topological features on the glass substrate and the nanoscale cellular adhesion apparatus [21]. In our prior work, this nanoroughened glass substrate was employed to recover cancer cells spiked in blood samples, in a fixed device setting, with capture efficiencies of over 90 % for different cancer cell lines [31]. Expanding on this proof-of-concept work, we hypothesized that further improvements in CTC capture performance and blood sample throughput could be achieved by using a confining microfluidic environment around the nanoroughened glass substrate to promote cell-substrate interactions for highly efficient CTC capture.

Herein we introduce our new microfluidic CTC capture platform and demonstrate its utility in recovering cancer cells with heterogeneous molecular properties and those obtained from two mouse models of cancer. Our microfluidic CTC capture platform integrates two functional components: 1) a RIE-generated nanoroughened glass substrate with nanoscale topological structures to enhance adherent interactions between the glass substrate and cancer cells, and 2) an overlaid polydimethylsiloxane (PDMS) chip with a low profile microfluidic capture chamber that promotes CTC-substrate contact frequency. In this work we showed that the microfluidic CTC capture chip could capture > 80 % of breast and lung cancer cells spiked in whole blood samples independent of the cell lines’ EpCAM expression. The microfluidic CTC capture chip also captured equally well A549 lung cancer cells in their epithelial- or mesenchymal-like state before and after transforming growth factor beta (TGF-β)-induced EMT. To further demonstrate the clinical utility of the microfluidic CTC capture chip, we collected whole blood from mice with breast cancer orthotopic xenografts and demonstrated excellent label-free CTC capture efficiency by the microfluidic CTC capture chip. More importantly, in a syngeneic mouse model of lung cancer utilizing cell lines with known metastatic and non-metastatic capabilities, CTCs were detected in all the mice with a detectable primary tumor independent of the metastatic propensity of the cell line implanted. This highlights the fact that not all CTCs are capable of forming and proliferating as metastases and our newly developed microfluidic CTC capture device is able to recover this less metastatically potent population as well.

## Methods

### Cell culture

MCF-7, MDA-MB-231, and A549 cells were acquired from ATCC and SUM149 cells were certified via short tandem repeat analysis (FTA barcode STR13871). 344SQ and 393P cell lines were derived from K-ras/p53 mutant mice as described in Gibbons et al. [32, 33]. MCF-7 cells were maintained in high-glucose DMEM (Invitrogen); MDA-MB-231, 344SQ, and 393P cells in RPMI-1640 (Invitrogen); SUM-149 cells in Ham’s F-12 w/L-glutamine (Fisher Scientific); and A549 cells in DMEM/F12 (Invitrogen). MCF-7, MDA-MB-231, and SUM-149 media containted 0.5 μg mL^−1^ Fungizone, 5 μg mL^−1^ Gentamicin, 100 units mL^−1^ penicillin, and 100 μg mL^−1^ streptomycin (all Invitrogen). Addtionally, SUM-149 cells were supplemented with 5 μg mL^−1^ Insulin and 1 μg mL^−1^ Hydrocortisone (both Sigma-Aldrich). A549, 344SQ, and 393P were supplemented with penicillin and streptomycin as above [32, 33]. All media contained 10 % fetal bovine serum (Atlanta Biological) except SUM-149 media which had 5 %. SUM-149 cells were maintained at 37 °C with 10 % CO_2_ and all other cell lines at 37 °C with 5 % CO_2_. Fresh 0.25 % trypsin-EDTA in phosphate buffered saline (PBS) was used to re-suspend cells. To induce the EMT, A549 cells were cultured with TGF-β at 5 ng mL^−1^ in serum free media for 72 h. TGF-β is a potent inducer of EMT [34–37].

### Chip fabrication

The microfluidic chip includes three components: a PDMS microfluidic chamber, an RIE-etched nanorough glass substrate, and a polyacrylate gadget to sandwich the chamber and substrate together. The microfluidic chamber was generated by replica molding using a Si mold fabricated using microfabrication. The detailed protocol for fabrication of the microfluidic CTC capture chip is described in the Additional file 1.

### Human blood specimens

Human blood specimens from healthy donors were collected in EDTA-containing vacutainers and were processed and assayed within 6 h of collection. RBC Lysis Buffer (eBioscience) was added to whole blood at a 10:1 *v*/*v* ratio. After incubation for 10 min at room temperature, the sample was diluted with 20–30 mL PBS to stop the lysing reaction and then centrifuged at 300 g for 10 min. After discarding the supernatant, the cell pellet was re-suspended in an equivalent volume of growth medium before use in CTC capture assays.

### Mouse models of cancer

Care of animals and experimental procedures were according to the University of Michigan University Committee on Use and Care of Animals (UCUCA) approved protocols #PRO5314 and #PRO4116. To generate breast cancer xenografts, 1 × 10^6^ MDA-MB-231 or SUM-149 cells were injected orthotopically into the left inguinal mammary fat pad of each female Ncr nude mouse (Taconic). The cells were suspended in 50 μL PBS and 50 μL Matrigel (Becton Dickinson). For the lung cancer studies, 1 × 10^6^ cells of two mouse lung cancer cell lines (metastatic 344SQ and non-metastatic 393P) with differential metastatic capability [32, 33] were subcutaneously implanted on either side of the dorsal flank in C57BL/6 mice (Taconic). Tumor growth was monitored weekly by caliper measurement with ellipsoid volumes calculated using ½ x length × width × height. Before euthanizing the mice, blood samples (0.3–0.8 mL) were collected via cardiac puncture under anesthesia to quantify CTCs.

### CTC capture from in vitro spiked blood samples

Prior to CTC capture assays, cancer cells were first labeled with CellTracker Green (Invitrogen) before mixed with Δ9-DiI-stained (Invitrogen) leukocytes in lysed blood. The total cancer cell number in the blood sample was first quantified using a hemocytometer before the spiked sample was diluted using lysed whole blood to achieve the desired final CTC concentration. For the capture of pre- and post-EMT A549 cells in admixture, pre- and post-EMT A549 cells were first labeled with CellTracker Green (Invitrogen) and CellTracker Blue (Invitrogen), respectively, before mixed in cell culture medium.

The CTC capture chip was assembled and connected to a custom-built pressure control setup. The PDMS microfluidic chamber was washed with PBS for 5 min before 1.0 mL of spiked blood sample was loaded at a flow rate of 200 μL min^−1^ and incubated for 30 min - 1 h at 37 °C with 5 % CO_2_. After the CTCs adhered, the chamber was washed with PBS then loaded with 4 % paraformaldehyde (PFA; Electron Microscopy Sciences) in PBS for 20 min to fix captured CTCs. The nanorough glass substrate was then detached from the PDMS chamber and rinsed with PBS to remove floating cells. Adherent cells immobilized on the nanorough glass substrate were then imaged directly using a fluorescence microscope (Nikon Eclipse Ti-S, Nikon) equipped with an electron multiplying charge-coupled device (EMCCD) camera (Photometrics). To quantify CTC capture yield, the entire glass surface area was scanned on a motorized stage (ProScan III, Prior Scientific). Image processing software ImageJ (National Institutes of Health) was used to determine the number of CTCs.

### CTC capture from in vivo mouse models

Capture of CTCs from mouse blood samples was performed using a procedure similar to the one employed for spiked blood samples. To visualize and quantify CTCs captured on the nanorough glass substrate, immunostaining was performed after the glass substrate was detached from the microfluidic chamber. After the PBS rinse as above, adherent cells were permeabilized with 0.25 % Triton X-100 (Roche Applied Science) in PBS for 10 min. Fixed cells were incubated with 10 % goat serum (Invitrogen) for 1 h before another 1 h incubation with primary antibodies to cytokeratin (FITC; BD Biosciences) and mouse CD45 (PE) and DAPI to identify cancer cells, leukocytes, and cell nuclei, respectively. CTCs were identified by: positive staining of anti-cytokeratin and DAPI; negative staining of anti-CD45; and appropriate morphometric characteristics including cell size, shape, and nuclear size. The researcher counting CTCs was blinded to the mouse group and tumor characteristics.

### Statistical analysis

Student’s two-sample, unpaired t-tests were calculated using GraphPad Prism software with *P*-values < 0.05 considered statistically significant.

## Results

### Capture of cancer cells independent of surface protein expression

**Fig. 1:**
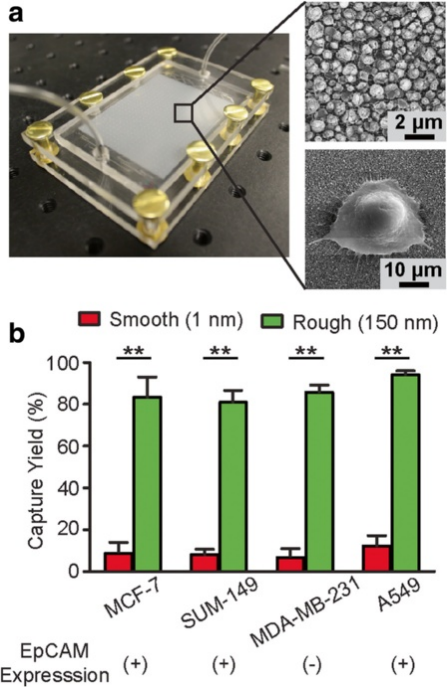
Nanotopography-based microfluidic chip for CTC capture. **a** Photo of the microfluidic CTC capture chip (*left*) and SEM images (*right*) showing the nanorough glass surface (*top right*, *R*_*q*_ = 150 nm) and a cancer cell adhered to the surface (*bottom right*). **b** Bar graph showing 30 min capture yield for breast cancer cells (MCF-7, MBA-MB-231, and SUM-149) and lung cancer cells (A549) using the capture chip with smooth (*R*_*q*_ = 1 nm) and nanorough (*R*_*q*_ = 150 nm) glass surfaces as indicated. For each cell type, 1,000 cells were spiked in 1 mL lysed human blood. EpCAM expression of each cell line is denoted below the graph. Error bars, s.e.m. (*n* = 4). **, *p* < 0.01

We have recently developed a simple yet precisely controlled method to generate random nanoroughness on glass surfaces using reactive ion etching (RIE) [38]. RIE-based nanoscale roughening of glass surfaces is consistent with a process of ion-enhanced chemical reaction and physical sputtering [39]. In our previous work, we have shown that bare glass surfaces treated with RIE for different periods of time can acquire different levels of roughness (as characterized by the root-mean-square roughness *R*_*q*_; *R*_*q*_ = 1–150 nm) with a nanoscale resolution (Additional file 1: Figure S1) [38]. To validate the efficiency of RIE-generated nanorough glass surfaces (Fig. 1a) for the capture of cancer cells with different surface protein expression, three breast cancer cell lines, MCF-7 (EpCAM-positive, or EpCAM+), SUM-149 (EpCAM+), and MDA-MB-231 (EpCAM-negative, or EpCAM-) [40–42] spiked in minute amounts in culture medium (1,000 cells in 1 mL medium) as single cells were injected into the microfluidic CTC capture chip with either a smooth glass surface (*R*_*q*_ = 1 nm) or a nanoroughened glass surface (*R*_*q*_ = 150 nm) for 30 min. Quantitative analysis revealed that the capture yield of cancer cells, defined as the ratio of the number of cancer cells captured on the glass surface to the total number of cells initially seeded, was 85.7, 80.9, and 86.5 % for MCF-7, SUM-149, and MDA-MB-231, respectively, for the nanorough glass surface with *R*_*q*_ = 150 nm (Fig. 1b). Additional passes of the spiked blood samples over the device did not increase yields further (data not shown). In distinct contrast, experiments using the smooth glass surface with *R*_*q*_ = 1 nm showed drastically lower capture yields for MCF-7, SUM-149, or MDA-MB-231 cells (6.7 % for MCF-7, 8.0 % for SUM-149, and 8.7 % for MDA-MB-231) (Fig. 1b). We further performed cell capture assays using the EpCAM+ A549 lung cancer cell line [43] and observed a similarly significant enhancement of cancer cell capture yield by the nanoroughened glass surface (Fig. 1b). Leukocyte capture yields were similar to our previously reported results [31]. Together, our results in Fig. 1 suggest a very strong propensity for cancer cells to adhere to RIE-generated nanorough glass surfaces regardless of the cells’ EpCAM expression status, and further support a superior efficiency of the label-free nanoroughened glass substrate for capturing CTCs.

### Capture of cancer cells before and after TGF-β-induced epithelial-mesenchymal transition

**Fig. 2:**
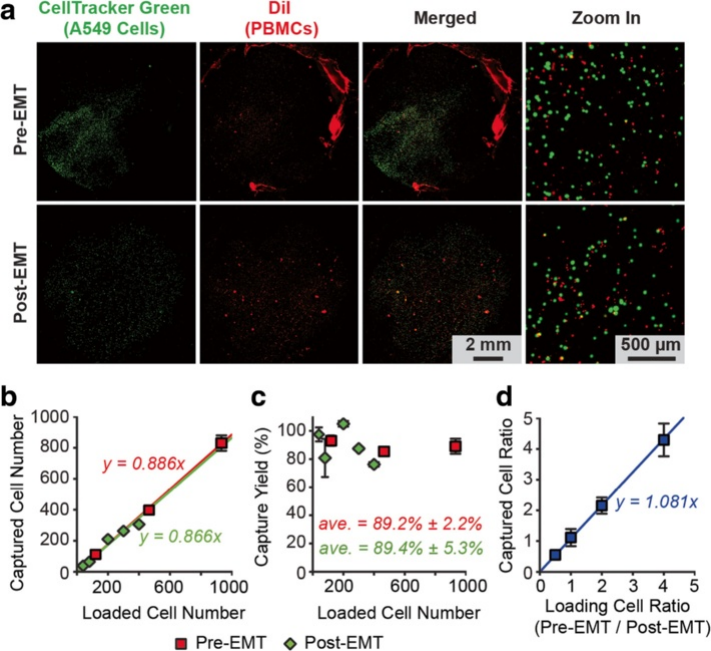
Capture of pre- and post-EMT lung cancer cells using the nanotopography-based microfluidic CTC capture chip. **a** Representative staining images showing pre- (*top*) and post-EMT (*bottom*) A549 cells captured on nanorough glass surfaces (*R*_*q*_ = 150 nm) 1 h after cell seeding. 10,000 pre- and post-EMT A549 cells labeled with CellTracker Green were spiked in 500 μL lysed blood that was pre-stained with DiI to label peripheral blood mononuclear cells (PBMCs). **b** & **c** Regression analysis of 1 h capture efficiency for pre- and post-EMT A549 cells (*n* = 40–900 spiked in 500 μL lysed blood) using the microfluidic CTC capture chip. The number of A549 cells captured (**b**) and the capture yield (**c**) is plotted as a function of the total number of A549 cells spiked in blood samples. **d** Ratio of pre- and post-EMT A549 cells captured 1 h after cell seeding as a function of their ratio when spiked in blood samples. 1,000 post-EMT A549 cells were mixed with 500–4,000 pre-EMT cells in 500 μL lysed blood to achieve ratios from 2 : 1 to 1 : 4. *Solid lines* in **b** & **d** represent linear fitting. Error bars, s.e.m. (*n* > 4)

Through the metastatic cascade, tumor cells are posited to undergo an EMT, which alters adhesive surface protein expression along with many other aspects of cellular behaviors [44, 45]. During this EMT, in addition to acquiring a migratory and invasive phenotype, tumor cells express mesenchymal proteins and concomitantly lose epithelial markers including the expression of EpCAM [46]. To demonstrate specifically that the capture of cancer cells by the RIE-generated nanorough glass substrate was independent of a cancer cell’s epithelial or mesenchymal state, we used the A549 cell culture model of TGF-β-induced EMT and spiked known quantities of pre- and post-EMT A549 cells (*n* = 40–10,000) into 500 μL lysed human blood (Fig. 2a). After culture with TGF-β for 72 h, A549 cells express significantly reduced levels of EpCAM mRNA (Additional file 1: Figure S2) [47]. Yet despite these lung cancer cells’ dynamic EpCAM expression, high capture yields were achieved when seeding the cells for 1 h in the microfluidic CTC capture chip with a nanoroughened glass surface (*R*_*q*_ = 150 nm) for both pre- and post-EMT A549 lung cancer cells, even at extremely low cancer cell concentrations (80 cells mL^−1^) (Fig. 2b & c). Strong linear correlations between the number of cancer cells captured vs. the number of cancer cells initially loaded (*n* = 40–900) were observed for both pre- and post-EMT A549 cells (Fig. 2b). Averaged across all cell concentrations assayed (80–20,000 cells mL^−1^), capture yields were 89.4 % ± 5.3 % for post-EMT A549 cells and 89.2 % ± 2.2 % for pre-EMT A549 cells (Fig. 2c, Additional file 1: Fig. S2). We further examined the effect of admixtures of pre- and post-EMT A549 cells on capture efficiency by varying the ratio of pre- and post-EMT A549 cells spiked in the same blood sample. Here 1,000 post-EMT A549 cells were mixed with 500–4,000 pre-EMT cells in 500 μL lysed blood to achieve a cell ratio from 2 : 1 to 1 : 4 (Fig. 2d). Cell capture assays using the microfluidic CTC capture chip for 1 h revealed that capture yield was not significantly affected by the relative proportions of pre- or post-EMT A549 cells with differing EpCAM expression and remained constant over the entire range of cell ratios of pre- and post-EMT A549 cells (Fig. 2d). Together, our results in Fig. 2 support that the RIE-generated nanorough glass surfaces can achieve efficient capture of CTCs independently of the cancer cell’s epithelial or mesenchymal state or EpCAM expression, demonstrating the applicability of the microfluidic CTC capture device for the capture and enumeration of rare tumor cells from heterogeneous cell samples and throughout a tumor’s metastatic progression, even in the setting of a dynamic EMT process.

### Capture of CTCs from a human breast cancer orthotopic xenograft mouse model

**Fig. 3:**
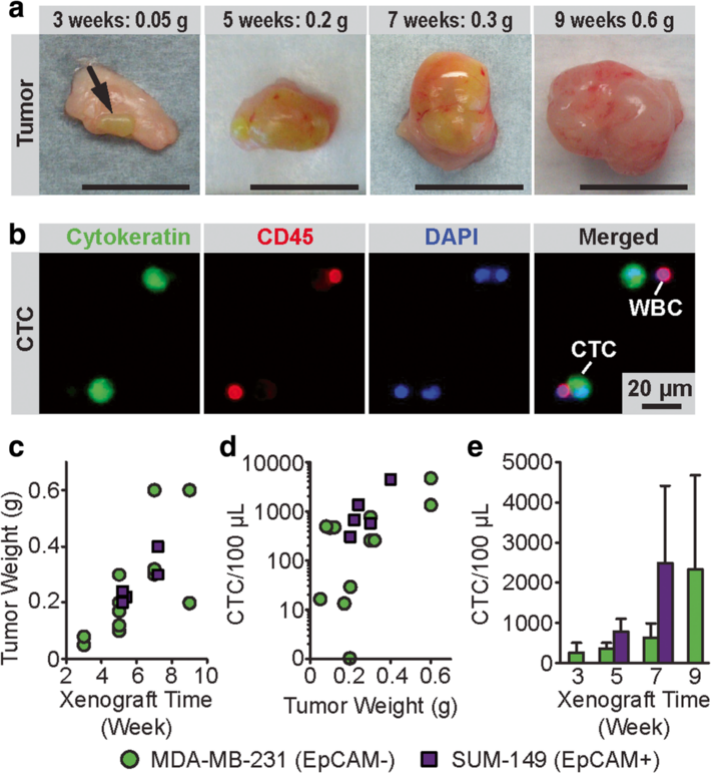
CTCs captured using the microfluidic CTC capture chip from mice with breast cancer orthotopic xenografts. **a** Photos of MDA-MB-231 xenografts, 1 cm scale bar. The arrow indicates the small tumor at 3 weeks. **b** Representative staining images showing CTCs captured on nanorough glass surfaces from mice with MDA-MB-231 tumor xenografts. Cells were co-stained for nuclei (DAPI; *blue*), cytokeratin (*green*), and CD45 (*red*). **c-e** Temporal changes in CTC number and tumor weight during tumor progression. Tumor weight (**c**) from mice with MDA-MB-231 and SUM-149 tumor xenografts as a function of xenograft time. Scatter plot (**d**) of CTC number per 100 μL blood vs. tumor weight. Bar plot (**e**) showing number of CTCs captured by the microfluidic CTC chip as a function of xenograft time. For each CTC capture assay, 300–800 μL blood samples were obtained via cardiac puncture. Error bars, s.e.m

We next assayed the microfluidic CTC capture chip with a nanoroughened glass surface (*R*_*q*_ = 150 nm) using an orthotopic xenograft mouse model of breast cancer. To generate tumor xenografts (Fig. 3a), 1 × 10^6^ MDA-MB-231 (EpCAM-) or SUM-149 (EpCAM+) breast cancer cells were injected into the left inguinal mammary fat pad of female Ncr nude mice [48]. When mice were euthanized to assess for tumor burden between 3 - 7 weeks of xenograft time, nearly the entire mouse blood volume (300–800 μL) was collected by cardiac puncture of the left ventricle from each mouse before assayed using the microfluidic CTC capture chip. CTCs, as defined by cytokeratin+, CD45-, DAPI+ staining (Fig. 3b), were successfully captured from 11 out of 12 mice bearing tumor xenografts of MDA-MB-231 cells and from all 5 mice with tumor xenografts of SUM-149 cells (Table 1). Data pooled from both EpCAM+ and EpCAM- breast cancer mouse models showed that the number of CTCs captured by the microfluidic CTC capture chip ranged from 13 to 4,664 cells per 100 μL of blood and increased drastically over the 9-week period during tumor progression, correlating positively with an increase in tumor weight (Fig. 3c-e).

**Table 1:** Capture of CTCs from mice with orthotopic breast cancer xenografts

Group	Sample	Xenograft time	End tumor weight (g)	Collected blood volume (μL)	Captured CTCs (CTCs/100 μL)
MDA-MB-231	#1	3 weeks	0.05	800	16
	#2		0.08	800	498
	#3	5 weeks	0.20	800	29
	#4		0.17	800	13
	#5		0.30	800	772
	#6		0.10	800	468
	#7		0.12	500	478
	#8	7 weeks	0.60	800	1348
	#9		0.30	800	259
	#10		0.32	800	261
	#11	9 weeks	0.20	800	0
	#12		0.60	800	4664
SUM-149	#13	5 weeks	0.22	300	675
	#14		0.20	800	306
	#15		0.24	700	1366
	#16	7 weeks	0.30	500	579
	#17		0.40	700	4408

MDA-MB-231 or SUM-149 xenografts of 1 × 10^6^ cells were grown before blood collection and enumeration of CTCs

### Capture of CTCs from metastatic and non-metastatic syngeneic mouse models of lung cancer

We next sought to assay the microfluidic CTC capture chip using a syngeneic mouse model of lung cancer. Two well-defined mouse lung cancer cell lines (344SQ and 393P) with different metastatic capabilities were subcutaneously implanted in a syngeneic host. Even though 344SQ and 393P lung cancer cells have distinct metastatic potential, both cell lines are derived from the same transgenic mouse model of lung cancer (p53 null, mutant Kras) [32, 33]. The 344SQ lung cancer cells form metastatic lesions from spontaneous and experimental metastatic assays (subcutaneous implantation and tail vein injection), whereas the 393P cell line does not metastasize by either assay [32]. However, both cell lines are capable of undergoing EMT in response to TGF-β with different kinetics and lose expression of epithelial markers [32, 33].

**Fig. 4:**
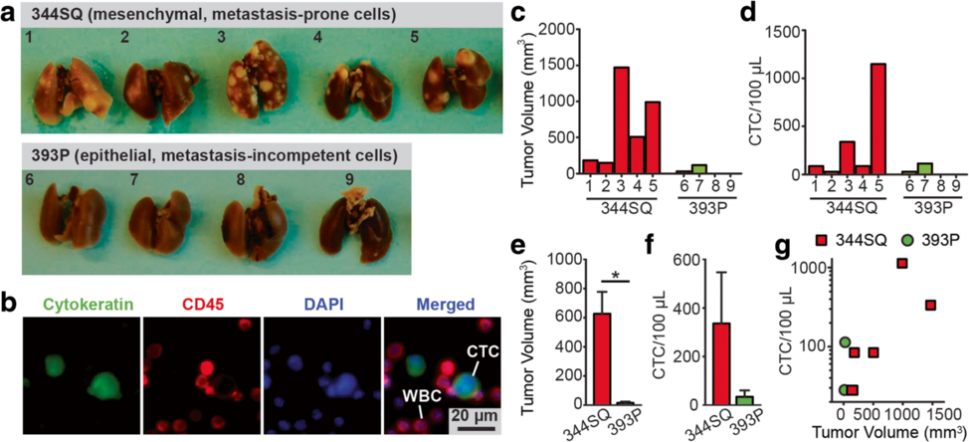
Capture of CTCs from metastatic and non-metastatic syngeneic mouse models of lung cancer. **a** Photos of lung metastases from 344SQ (*top*) and 393P (*bottom*) implants. Mouse 344SQ lung cancer cells are highly metastatic, while mouse 393P lung cancer cells are metastasis-incompetent. **b** Representative staining images showing CTCs captured on nanorough glass surfaces from mice implanted with 344SQ cells. Cells were co-stained for nuclei (DAPI; *blue*), cytokeratin (*green*), and CD45 (*red*). **c-g** Analysis of CTC number and tumor volume for mice with 344SQ and 393P tumor allografts. Bar plots show tumor volume (**c**) and CTC number per 100 μL blood (**d**) for individual mice. Bar plots showing average tumor volume (**e**) and average CTC number per 100 μL blood (**f**) of all mice. Scatter plot (**g**) of CTC number per 100 μL blood vs. tumor volume for mice with 344SQ and 393P tumor allografts. Mice were subcutaneously implanted with tumor allografts of 344SQ and 393P lung cancer cells. For each CTC capture assay, 350–600 μL blood samples were obtained via cardiac puncture. Error bars, s.e.m. *, *p* < 0.05

After 6 weeks of subcutaneous tumor growth, mice were sacrificed and whole blood was collected via cardiac puncture before being processed with the microfluidic CTC capture chip with a nanoroughened glass surface (*R*_*q*_ = 150 nm) (Additional file 1: Figure S3). Simultaneously, primary tumor volumes were measured and lungs were examined grossly for metastasis (Fig. 4a). The 344SQ primary tumors grew significantly larger and shed more CTCs than metastasis-incompetent 393P tumors (Fig. 4c-f). Using the microfluidic CTC capture chip, CTCs were detected in all five mice implanted with the metastatic 344SQ cell line (Fig. 4d, Table 2). Similar to results from the breast cancer xenograft model, the number of CTCs detected using the microfluidic CTC capture chip showed a positive correlation with primary tumor size (Fig. 4g). As expected, neither of the two mice implanted with the metastasis-incompetent 393P lung cancer cell line that formed palpable primary tumors (mice #6 and 7) had detectable metastatic lesions on their lungs (Fig. 4, Table 2). Surprisingly, however, we detected the presence of CTCs in all the mice, including those mice with metastasis incompetent 393P implants, with palpable primary tumors (Fig. 4d). This observation clearly demonstrates that the presence of CTCs alone may not be indicative of the presence of metastatic disease.

**Table 2:** Capture of CTCs from metastatic and non-metastatic syngeneic mouse models of lung cancer

Group	Sample	End tumor volume (mm^3^)	Collected blood volume (μL)	Captured CTCs (CTCs/100 μL)
Metastasis-Prone (344SQ)	#1	179	500	84
	#2	144	500	28
	#3	1470	350	336
	#4	503.5	500	84
	#5	988	500	1148
Metastasis-Incompetent (393P)	#6	15.8	400	28
	#7	40	350	112
	#8	No tumor	500	0
	#9	No tumor	600	0

The metastasis-prone 344SQ or metastasis-incompetent 393P lung cancer cell lines were subcutaneously implanted into mice that were sacrificed 6 weeks after implantation with blood collected for circulating tumor cell quantification

## Discussion

In this work, we have successfully developed a microfluidic CTC capture chip utilizing an RIE-generated nanorough glass surface as the substrate for efficient capture of CTCs regardless of cell size or surface protein expression. The microfluidic flow chamber incorporated on top of the nanorough glass surface promotes greater adhesive interactions of cancer cells with the nanorough glass substrate, thereby providing an effective strategy to achieve superior CTC capture efficiency. Other efforts that have been undertaken to isolate CTCs have primarily depended on either physical size differences between cancer cells and hematocytes or on the surface protein expression of either cancer cells or leukocytes [14–24, 49, 50]. In contrast, our CTC capture strategy leverages the differential adhesion preference to the RIE-generated nanorough glass surfaces between cancer cells and normal blood cells [31]. Mechanical properties of cancer cells represent a point of convergence in the metastatic cascade whereby only those cells within a tumor behaving in a precise biomechanical manner will successfully intravasate into the bloodstream. Since the mechanical phenotype of a cancer cell is the culmination of an array of heterogeneous factors both cell intrinsic and cell extrinsic [27, 30], we posit that using a CTC capture system that is mechanically focused and adhesion-based will have greater success in detecting CTCs with different molecular signatures. This fact was supported by this present study as our adhesion-based microfluidic CTC capture chip was capable of capturing heterogeneous CTC populations independent of their EpCAM expression status or phenotypic state along the epithelial-mesenchymal continuum. Specifically, with the microfluidic CTC capture device, we were able to achieve capture yields of > 80 % for both EpCAM+ (MCF-7, SUM-149, A549) and EpCAM- (MDA-MB-231) cancer cell lines spiked in whole blood samples. While our present system relied upon the fixation and staining of cells to verify CTC capture, the process could easily be altered to forgo this step instead detach viable cells for downstream single-cell sequencing studies and analysis. Furthermore, the microfluidic CTC capture device attained high capture yields for both pre- and post-EMT lung cancer cells – and with equal affinity – in an in vitro model of induced EMT. Unbiased efficient capture of heterogeneous populations of CTCs regardless their EpCAM expression status is important, as EpCAM expression in tumor cells varies between patient to patient and within a patient over time as it is rapidly down-regulated during EMT. Similarly, many other surface markers on cancer cells are dynamically expressed over the course of tumor dissemination and the metastatic cascade [9–11, 51, 52]. Therefore, the precise surface marker expression of CTCs is a moving target during tumor progression, requiring capture methods targeting the whole CTC population to be independent of CTCs’ surface marker expression.

Although there are several other microfluidic platforms capable of achieving high CTC capture efficiency, many of them depend on the use of positive selection agents (i.e. anti-EpCAM antibody or aptamer) [6, 8, 53, 54]. These methods inherently require a priori assumption about the surface protein expression of CTCs that have been proven to be a dynamic and inconsistent population [6, 8]. Some tumor cells may shed from the primary tumor and enter the bloodstream after undergoing the EMT process and losing their epithelial properties [45, 55]. It has been proposed that the EMT process may additionally cause a series of other CTC feature changes apart from the loss of epithelial properties, such as enhanced invasiveness and elevated resistance to apoptosis [56]. In agreement with this, a recent study has revealed dynamic changes of epithelial and mesenchymal compositions of CTCs with disease progression among patients with breast cancer [9]. Together, it is clear that some CTCs may experience phenotypic changes during tumor evolution and that the expression of EpCAM may be transient, so EpCAM expression based methods may potentially miss a substantial subset of CTCs [57, 58]. Thus, any positive marker-based selection method can bias captured CTCs toward a population that is not representative of the CTCs in a patient [8, 59]. The limited number of CTCs detected in patients even in late stages of metastases may well be a result of the use of CTC detection methods that heavily rely on EpCAM expression by CTCs [60–62]. New methods, like the microfluidic CTC capture chip using the label-free nanoroughened glass substrate, are critically needed to capture the entirety of heterogeneous CTC populations. In this work we have shown that by focusing on a biomechanical property dependent on a multitude of cellular signals, we can capture CTCs in different morphologic states and irrespective of EpCAM expression, thus our adhesion-based microfluidic CTC capture is marker and molecular independent.

To advance the clinical relevance of our microfluidic CTC capture chip further, we studied two in vivo models of breast and lung cancer. In orthotopic xenografts of EpCAM+ and EpCAM- breast cancer cell lines, clear correlations between tumor size and CTC number were observed for both MDA-MB-231 and SUM-149 xenografts, supporting the independence of our CTC capture methodology from cell surface marker expression. Our adhesion-based method for capturing heterogeneous CTC populations was further demonstrated by the use of a syngeneic lung cancer mouse model with differential metastatic capabilities. In this model, a positive correlation between primary tumor size and CTC number was observed. Interestingly, CTCs were also detected by our microfluidic CTC capture chip in two mice implanted with the non-metastatic 393P cell line. These mice did not grow overt lung metastases as did all the mice in the metastatic 344SQ cell line cohort. Thus, a population of CTCs incapable of forming metastases was detected by the microfluidic CTC capture chip, supporting that cellular signals and biological processes that allow for individual cell invasion and intravasation are not identical to those governing the seeding of fruitful metastases. It is important to understand the differences in the nature of these CTCs to determine their true significance in patient prognosis and in the clinical management of cancer, and our microfluidic CTC capture chip allows for both populations’ study with its unbiased capture method based on the selective adhesion of cancer cells.

## Conclusions

In summary, we have developed a promising strategy for broad-based CTC capture by exploiting the preference of cancer cells to adhere to RIE-generated, nanoroughened glass surfaces that is independent of CTCs’ marker expression or epithelial-mesenchymal state, both of which change throughout the development of a tumor and metastases. We show that our microfluidic CTC capture method is broadly applicable to capturing heterogeneous CTC populations in vitro and in two animal models of cancer, demonstrating its potential to collect highly diverse CTC subgroups. Future exploration of the molecular and functional differences in different subpopulations of CTCs captured with our nanoroughened methodology will yield insights into their differential effects on tumor progression and outcomes.

## Abbreviations

CTC, circulating tumor cell; EMT, epithelial-to-mesenchymal transition; EpCAM, epithelial cell adhesion molecule; PBMS, peripheral blood mononuclear cells; PBS, phosphate buffered saline; PDMS, polydimethylsiloxane; RBC, red blood cell; SEM, scanning electron microscope; TGF-β, transforming growth factor beta; UCUCA, University Committee on Use and Care of Animals

## Additional file

Additional file 1: Supporting Materials: Additional Methods and Supporting Figures. (PDF 688 kb)
