# Supplementary material for: Nanoroughened adhesion-based capture of circulating tumor cells with heterogeneous expression and metastatic characteristics
Source: BMC Cancer. 2016 Aug 8;16:614. doi: 10.1186/s12885-016-2638-x (PMC4977622; doi:10.1186/s12885-016-2638-x)
Supplement: Additional file 1: — Supporting Materials: Additional Methods and Supporting Figures. (PDF 688 kb) [file 12885_2016_2638_MOESM1_ESM.pdf]

Supporting Materials:

Additional Methods and Supporting Figures

1. Methods.

Fabrication of CTC capture chip. The CTC capture chip includes three components: a PDMS microfluidic chamber, a patterned nanorough glass substrate, and a polyacrylate gadget sandwiching the PDMS chamber and the patterned nanorough glass substrate. The glass substrate has dimensions of 50 mm × 76 mm and an effective nanoroughed region of 44 mm × 56 mm. The PDMS microfluidic chamber (height 400 μm, width 44 mm, length 56 mm) was produced by soft-lithography using a replicate on a silicon mold. Briefly, a silicon master for the microfluidic chamber was fabricated using photolithography and deep reactive ion etching (DRIE; STS Deep Silicon Etcher, Surface Technology Systems). The silicon master was then silanized with (tridecafluoro-1,1,2,2,-tetrahydrooctyl)-1-trichlorosilane vapor (United Chemical Technologies) for 4 hr under vacuum to facilitate subsequent release of the PDMS microfluidic chamber from the silicon master. PDMS prepolymer (Sylgard 184, Dow-Corning) was then prepared by thoroughly mixing the monomer with the curing agent (at a *w*/*w* ratio of 10:1), poured onto the silicon master and cured at 110 ºC for 1 hr. The fully cured PDMS chamber was peeled off from the silicon mold and the excess PDMS was trimmed using a razor blade and two through-holes were punched at the inlet and outlet for the tubing connections.

For the patterned nanorough glass substrates, a photoresist was first spin-coated on glass wafers (Borofloat 33, Plan Optik) and patterned using photolithography. The glass wafer was then processed with RIE (LAM 9400, Lam Research) for different periods of time to generate nanoscale surface roughness (ranging from 1 nm to 150 nm) on the open regions of the glass wafer, where the photoresist had previously been developed and dissolved. The RIE process condition was selected as: SF_6 (8 sccm), C_4F_8 (50 sccm), He (50 sccm), Ar (50 sccm), chamber pressure (1.33 Pa), bias voltage (100 V), and radio frequency power (500 W), with the resulting glass etch rate as about 50 nm min^{-1}. After the RIE process, the photoresist was striped using solvents, and the glass wafer was cleaned using distilled water. The glass wafers were then cut into the designated size (50 mm × 76 mm) using an ADT7100 dicing saw (Advanced Dicing Technologies Ltd.).

To assemble the chip, a device holder composed of two polyacrylate plates was machined to sandwich the PDMS microfluidic chamber and the nanorough glass substrate using screws at the four corners and along the edges of the polyacrylate plates. Two through-holes were drilled on the top polyacrylate plate to align with the inlet and outlet holes of the PDMS microfluidic channel, thus allowing a convenient tubing connection to the microfluidic chamber. The complete assembly using the polyacrylate plates to hold the PDMS microfluidic chamber could withstand a pressure of about 50 psi without leaking.

SEM Specimen Preparation. Cell samples were washed three times with 50 mM Na-cacodylate buffer (pH 7.3; Sigma-Aldrich), fixed for 1 hr with 2% glutaraldehyde (Electron Microscopy Sciences, Hatfield, PA) in 50 mM Na-cacodylate buffer, and dehydrated in a graded series of ethanol concentrations through 100% over a period of 1.5 hr. Dehydration in 100% ethanol was performed three times. Afterwards, dehydrated substrates were dried with liquid CO_2 using a super critical point dryer (Samdri®-PVT-3D, Tousimis, Rockville, MD). Samples were mounted on stubs, sputtered with gold palladium, observed and photographed under a Hitachi SU8000 Ultra-High Resolution SEM machine (Hitachi High Technologies America, Inc., Pleasanton, CA).

Surface Characterization Using Atomic Force Microscope. Nanoroughness of the glass surfaces was measured at room temperature with the Veeco NanoMan Atomic Force Microscope (AFM, Digital Instruments Inc., Santa Barbara, CA) using non-contact, tapping mode and standard Si tapping mode AFM tips with a scan rate of 1 Hz. The resulting map of the local surface height was represented using AFM topographs. The nanoroughness of each glass sample was characterized by the root mean square (RMS) roughness R_q of the local surface height over the scanned areas collected using the AFM topographs.

2. Supporting Figures

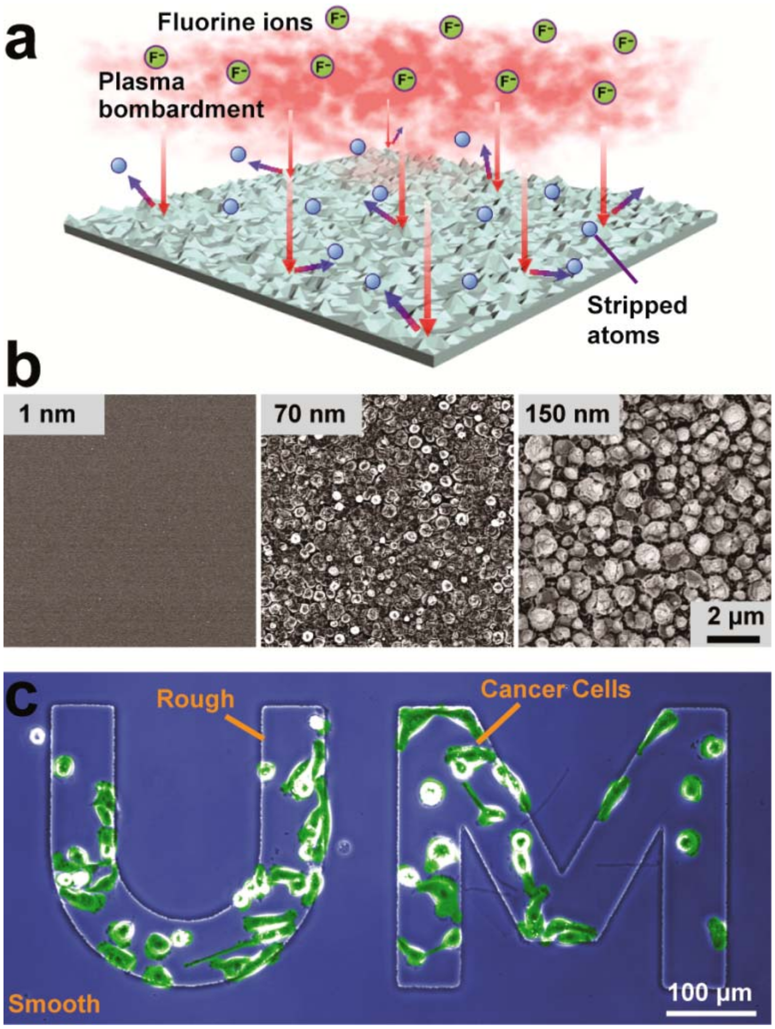

Figure S1. Intrinsic nanotopological sensing for CTC capture. (**a**) Schematic of nanotopography generated by RIE on glass surfaces. (**b**) SEM images of glass surfaces with their RMS nanoroughness (R_q) indicated. (**c**) Phase-contrast micrograph showing MDA-MB-231 cells selectively adhering to patterned nanorough letters (UM; R_q = 70 nm) on the glass surface 24 hr after cell seeding.

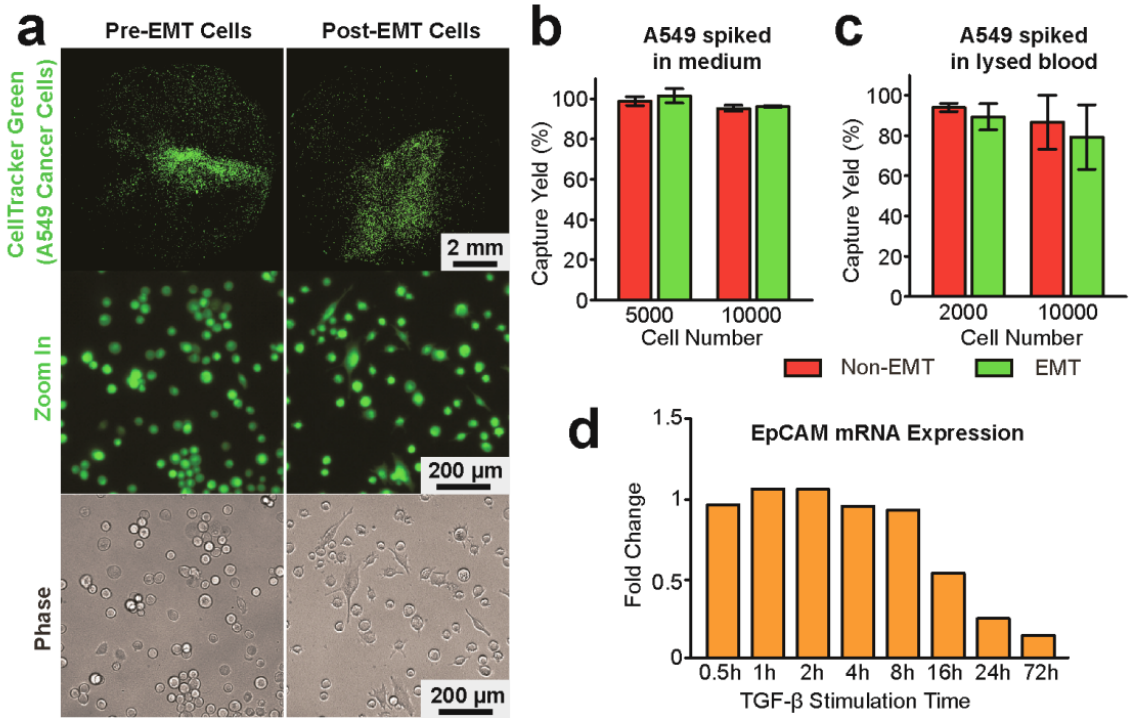

Figure S2. Capture of pre-EMT and post-EMT lung cancer cells spiked in cell culture medium or lysed mouse blood. (**a**) Representative fluorescence images and zoom-in fluorescence and phase imgages showing known quantities (10,000) of pre-EMT and post-EMT cancer cells as indicated spiked in cell culture medium captured on nanorough glass surfaces (R_q = 150 nm) 1 hr after cell seeding. Target cancer cells were labeled with CellTracker Green before capture. (**b**) Capture yields of pre-EMT and post-EMT cancer cells in cell culture medium on nanorough glass surfaces (R_q = 150 nm) 1 hr after cell seeding. (**c**) Capture yields of pre-EMT and post-EMT cancer cells in lysed blood on nanorough glass surfaces (R_q = 150 nm) 1 hr after cell seeding. (**d**) Time-course of A549 EpCAM mRNA expression during TGF-□-induced EMT as assessed by microarray analysis using an Affymetrix U133 plus chip. Fold change shown relative to unstimulated cells. Error bars, s.e.m. (n > 4).

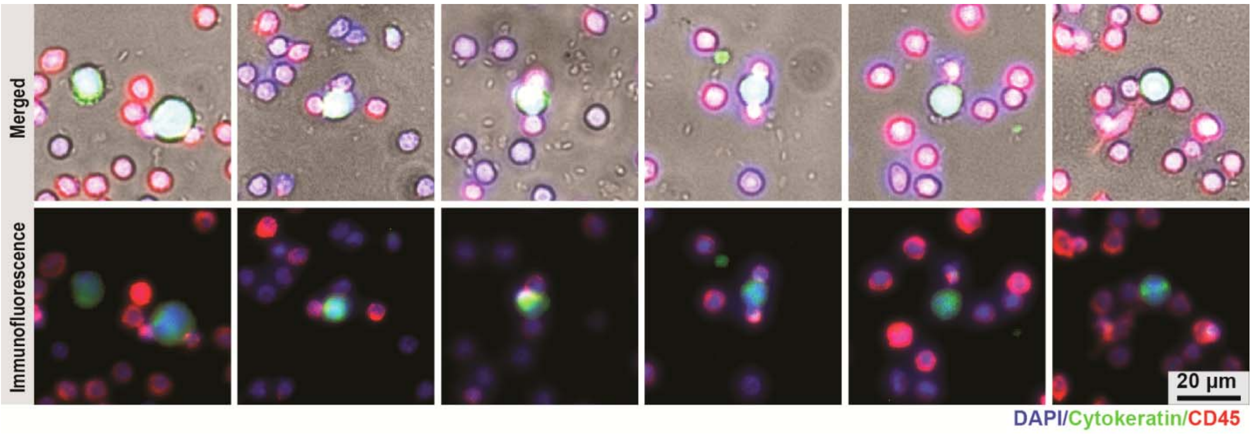

Figure S3. Representative merged immunofluorescence and phase images of captured CTCs from mice with 344SQ lung tumor allografts. Cells were co-stained for nuclei (DAPI; *blue*), cytokeratin (*green*), and CD45 (*red*).
